# Key candidate genes for male sterility in peppers unveiled via transcriptomic and proteomic analyses

**DOI:** 10.3389/fpls.2024.1334430

**Published:** 2024-02-06

**Authors:** Shimei Yang, Xirong Luo, Jing Jin, Ya Guo, Lincheng Zhang, Jing Li, Shuoqiu Tong, Yin Luo, Tangyan Li, Xiaocui Chen, Yongjun Wu, Cheng Qin

**Affiliations:** ^1^ Industrial Technology Institute of Pepper, Key Laboratory of Plant Resource Conservation and Germplasm Innovation in Mountainous Region (Ministry of Education), College of Life Sciences/Institute of Agro-bioengineering, Guizhou University, Guiyang, China; ^2^ Engineering Research Center of Zunyi Pepper Germplasm Resources Conservation and Breeding Cultivation of Guizhou Province, Department of Modern Agriculture, Zunyi Vocational and Technical College, Zunyi, China; ^3^ Key Lab of Zunyi Crop Gene Resource and Germplasm Innovation, Zunyi Academy of Agricultural Sciences, Zunyi, China

**Keywords:** pepper, proteomics, transcriptomic, male sterility, *CaPRX* genes, crop breeding

## Abstract

This study aimed to enhance the use of male sterility in pepper to select superior hybrid generations. Transcriptomic and proteomic analyses of fertile line 1933A and nucleic male sterility line 1933B of *Capsicum annuum* L. were performed to identify male sterility-related proteins and genes. The phylogenetic tree, physical and chemical characteristics, gene structure characteristics, collinearity and expression characteristics of candidate genes were analyzed. The study identified 2,357 differentially expressed genes, of which 1,145 and 229 were enriched in the Gene Ontology and Kyoto Encyclopedia of Genes and Genomes databases, respectively. A total of 7,628 quantifiable proteins were identified and 29 important proteins and genes were identified. It is worth noting that the existence of *CaPRX* genes has been found in both proteomics and transcriptomics, and 3 *CaPRX* genes have been identified through association analysis. A total of 66 *CaPRX* genes have been identified at the genome level, which are divided into 13 subfamilies, all containing typical *CaPRX* gene conformal domains. It is unevenly distributed across 12 chromosomes (including the virtual chromosome Chr00). Salt stress and co-expression analysis show that male sterility genes are expressed to varying degrees, and multiple transcription factors are co-expressed with *CaPRX*s, suggesting that they are involved in the induction of pepper salt stress. The study findings provide a theoretical foundation for genetic breeding by identifying genes, metabolic pathways, and molecular mechanisms involved in male sterility in pepper.

## Introduction

Pepper (*Capsicum annuum* L.), an annual or perennial herb in the Solanaceae family, is native to the tropical and subtropical regions of Central and South America. It is cultivated globally and is a major vegetable species, especially in China (Wu et al., 2013) where Guizhou province has the largest pepper cultivation and processing area. Its products are distributed nationwide, rendering pepper cultivation a cornerstone industry in Guizhou (Liu et al., 2022). Consequently, pepper breeding has gained increased importance, and thus, it is imperative to develop new pepper germplasms, improve strain selection, and enhance breeding technology. With continuous research in pepper breeding, genes related to nuclear male sterility (NMS) and cytoplasmic male sterility (CMS) have been gradually discovered in recent years. Male sterility is abundant in flowering plants, offering a convenient pathway for the breeding of new food and economic crops. Presently, common crops such as corn, rice, and wheat rely exclusively on crossbreeding to provide growers with seed resources and maximize product quality and economic returns (Rajput and Kandalkar, 2018; Sadimantara et al., 2018; Lu et al., 2020). To develop high-quality germplasm resources and breeding methods for pepper varieties, efficient selection and breeding techniques are essential. Two primary methods for propagating hybrid pepper seeds include manual emasculation and manual pollination using male sterile lines (Lv et al., 2020). The latter is more efficient as it eliminates the need for manual emasculation, thereby enhancing purity and reducing hybrid seed costs. However, further research is required to enhance the use of male sterility in pepper to select elite hybrid generations.

The genetic control of male sterility can occur via CMS or NMS, also known as genetic male sterility (GMS) (Morales et al., 2022). CMS, a condition under which a plant is unable to produce functional pollen, is widespread among higher plants (Eckardt, 2006). However, NMS is caused by a defect in a nuclear gene, and it is usually inherited as a recessive trait (Budar and Pelletier, 2001). Meanwhile, NMS is important for understanding microspore development, and it could be used to facilitate the development of new strategies to control male sterility (Chen et al., 2019). In recent years, many key genes related to infertility have been precisely located and discovered. Wang et al. (2019) found that orf300a and orf314a were strong candidate genes for CMS in pepper via mitochondrial assembly of the sterile line 138A and maintainer line 138B. Another study documented more than 20 GMS mutants in pepper (Dong et al., 2022). Transcriptomic analysis of sterile (160S-male sterile (MS) and fertile (160S-MF) flowers of *Brassica napus* revealed that heat shock proteins, antioxidants, skeletal proteins, GTPase, and calmodulin might be involved in thermosensitive GMS (Tang et al., 2019). However, pollen sterilization is critical for male sterility. Jacobowitz et al. found that PRX9 and PRX40 affect the development of normal anthers of *Arabidopsis thaliana*. Using immunochemical and biochemical methods, the researchers demonstrated that PRX9 and PRX40 can crosslink extension proteins to promote the integrity of the tapete wall during anther development (Jacobowitz et al., 2019).

Peroxidase is an oxidoreductase that is widely distributed in nature and can facilitate the oxidation of various organic and inorganic substrates using hydrogen peroxide and related compounds (de Oliveira et al., 2021). In addition to animal peroxidases, hemoglobin peroxidases are categorized into three classes based on their sequence and catalytic properties: Class I, II, and III peroxidases (Yang et al., 2020). Peroxidases are further classified into three major types based on their protein structures and catalytic properties: Class I (ascorbate peroxidase), Class II (lignin peroxidase), and Class III (secretory peroxidase) (Bhatt and Tripathi, 2011). Class III peroxidases (PRXs, EC 1.11.1.7) are plant-specific enzymes that have been extensively studied in higher plants, particularly in terms of growth, development, and responses to various stresses such as salt, drought, and metal toxicity (Almagro et al., 2008; Chen et al., 2022). PRXs are widely distributed in the plant kingdom and have been reported in various plant groups, including Chlorophyta, Euglenophyta, Rhodophyta, Byophyta, Pteridophyta, and all the Spermatophyta studied to date (Passardi et al., 2007). Notably, 73 PRXs have been identified in *Arabidopsis thaliana* (Tognolli et al., 2002), 138 in *Oryza sativa* (Passardi et al., 2004), and 374, 159, and 169 in wheat (*Triticum aestivum*, *Triticum urartu*, and *Aegilops tauschii*, respectively) (Yan et al., 2019). Additionally, 102 have been identified in *Solanum tuberosum* and 210 in *Nicotiana tabacum* (Yang et al., 2020; Cheng et al., 2022). Increasingly, PRX genes were identified in multiple species, indicating their involvement in plant growth and development and their important roles in stress response.

Type III plant peroxidases (EC 1.11.1.7) are well-recognized for their crucial roles in various metabolic reactions during plant defense processes. These peroxidases are involved in a wide range of physiological processes, including lignification, suberization, auxin metabolism, crosslinking of cell wall proteins, defense against pathogen attacks, salt tolerance, and response to oxidative stress (Hiraga et al., 2001; Almagro et al., 2008; Pandey et al., 2017). Research has shown that *OsPRX38* overexpression in transgenic *Arabidopsis* can enhance arsenic tolerance and increase lignification and cell wall–associated peroxidase activity in a positive correlation (Kidwai et al., 2019). In addition, it can enhance cold tolerance in overexpressing plants (Kim et al., 2012) and play a role in regulating plant hypocotyl elongation and auxin levels (Cosio et al., 2009). Furthermore, the Arabidopsis CIII PRX gene *AtPRX71* is strongly expressed during the loss of cell wall integrity, affecting growth and cell size and positively regulating reactive oxygen species levels (Raggi et al., 2015).

Herein, a combination of proteomics and transcriptomics analysis was used to identify key genes in MS pepper lines by comparing the differential expression of proteins and genes. In addition, the study examined the differentially expressed gene, Gene Ontology (GO) and Kyoto Encyclopedia of Genes and Genomes (KEGG) enrichment, differential metabolic pathways, and protein interactions in the sterile and fertile pepper lines. Furthermore, a systematic identification analysis of the key gene *CaPRX* was conducted to assess its structural characteristics, physicochemical properties, and collinearity. Finally, this study explored the network diagram and expression pattern of the *CaPRX* gene under salt stress conditions. By integrating transcriptome and proteome analysis of nucleic MS line and FL of pepper, candidate genes of male sterile line of capsicum were preliminarily identified. Thus, the study findings provide valuable insights into the selection and breeding of new pepper varieties and paves the way for further research in pepper breeding.

## Materials and methods

### Plant materials and processing

The fertile line (FL) (1933A) and nucleic male sterile line (NMSL) (1933B) of pepper were cultivated at the pepper-planting site of Zunyi Vocational and Technical College (Zunyi, Guizhou, 107°045’ E, 27°710’ N). Plants uniform in stage and height were selected. Flower buds (When the corolla is close to the dehiscent bud stage, peduncle 2–3 cm, corolla 2–3 cm. Buds, peduncle length 1–2 cm, transverse stem 0.5–0.8 cm, vertical stem 1–1.5 cm) from the FL 1933A and NMSL 1933B were collected in triplicate. Immediately after collection, the samples were stored in liquid nitrogen at −80°C for preservation.

### Transcriptome sequencing

The complete genome of pepper (*C. annuum* L., Zunla-1) was obtained from NCBI (GenBank accession: GCA_000710875.1) (Qin et al., 2014). The pepper transcriptome data were filtered using Trimmomatic-0.39 software (Bolger et al., 2014). The genome index was constructed using Hisat2-2.2.1 software, which facilitated the alignment of the transcriptome to the reference genes in pepper (Kim et al., 2019). FeatureCounts v2.0.3 software was used to compute the expected number of fragments per kilobase of transcript sequence per million base pairs sequenced for each gene in each sample (Liao et al., 2014). Lastly, DESeq2 software was used to analyze the differentially expressed genes between samples, with a threshold at *padj* of <0.05 and |log_2_ FC| of >1 (Love et al., 2014).

### Protein analysis

The FL 1933A and NMSL 1933B were lysed using SDT (4% [w/v] sodium dodecyl sulfate, 100 mM Tris/HCl, pH 7.6, and 0.1 M DTT) for total protein extraction. Protein quantification was performed using the BCA assay. A specific amount of protein from each sample was collected and subjected to trypsinization via the filter-aided proteome preparation method. The peptides were desalted using a C18 cartridge, lyophilized, resolubilized in 40 μL 0.1% formic acid solution, and quantified (OD_280_). The peptides (100 μg) from each sample were collected and labeled using a TMT labeling kit (Thermo Fisher Scientific, Waltham, MA, USA) as per the manufacturer’s instructions. The samples were divided into two groups, each containing three biological replicates, totaling six samples.

Each sample was separated using an EASY nLC nanoflow high-performance liquid chromatography liquid phase system. Buffer A comprised a 0.1% formic acid aqueous solution and buffer B comprised a 0.1% formic acid/acetonitrile aqueous solution (84% acetonitrile). The chromatographic column was equilibrated using 95% buffer A. The samples were loaded onto the loading column (Acclaim PepMap100, 100 μm × 2 cm, nanofiber C18, Thermo Fisher Scientific) via an autosampler and passed through an analytical column (EASY column, 10 cm, ID 75 μm, 3 μm, C18-A2, Thermo Fisher Scientific) at a flow rate of 300 nL/min.

The samples were then separated using chromatography and analyzed via mass spectrometry using a Q-exactive mass spectrometer. The detection mode was positive ion, parent ion scan range was 300–1800 m/z, resolution of the primary mass spectrum was 70,000 at 200 m/z, automatic gain control target was 1e6, maximum IT was 50 ms, and dynamic exclusion time was 60.0 s. The mass-to-charge ratios of the peptides and peptide fragments were collected as follows: following each full scan, 20 fragmentation profiles (MS2 scan) were collected; MS2 activation type was higher-energy collision dissociation; isolation window was 2 m/z; secondary mass spectral resolution was 17,500 at 200 m/z; normalized collision energy was 30 eV; and underfill was 0.1%.

### Transcriptome and proteome bioinformatics analyses

Protein clustering analysis was conducted using the Complexheatmap R package, which classified samples and protein expression into two dimensions (distance algorithm: Euclid, linkage: average linkage), yielding a hierarchical clustering heat map (Gu and Hübschmann, 2022). The subcellular localization of the proteins was predicted using CELLO (http://cello.life.nctu.edu.tw/) (Yu et al., 2004). The structural domains of the proteins were analyzed using Pfam data and compared with the InterProScan software package (Jones et al., 2014; El-Gebali et al., 2019). The target protein set was annotated for GO using Blast2GO and for KEGG using KAAS software (Conesa et al., 2005; Moriya et al., 2007). Enrichment analysis for GO and KEGG was performed using ClusterProfiler (Wu et al., 2021). The interaction relationships, direct and indirect, between the target proteins were identified using either the STRING (http://string-db.org/) databases. These interaction networks were generated and analyzed using Cytoscape v3.2.1 (Otasek et al., 2019).

### Family identification and analysis

The whole-genome data (v2.0) of *C. annuum* L. (Zunla-1 and Chiltepin) were sourced from NCBI (GenBank: GCA_000950795.1) (Qin et al., 2014). The *AtPRXs* gene of *Arabidopsis* was downloaded from the Arabidopsis website (http://www.arabidopsis.org/index.jsp) with the sequence referenced from a study by Yang et al. (Yang et al., 2022). The *PRXs* model file (PF00141) was obtained from the Pfam database (http://pfam.xfam.org/) and used for model searching via HMMer v3.3.2 (El-Gebali et al., 2019). A Blastp comparison was performed on 73 *Arabidopsis* sequences with an E-value of e-20 using MEGA X, (https://megasoftware.net/), and an neighbor-joining phylogenetic tree was constructed using a bootstrap value of 1000 and default parameters (Kumar et al., 2018). The identified *CaPRX* gene family was visualized using iTol (Letunic and Bork, 2021). The physicochemical properties of the pepper CaPRXs protein were analyzed using Expasy-ProtParam (https://web.expasy.org/protparam/). The conserved domains of the proteins were analyzed using NCBI CDD with default parameters (https://www.ncbi.nlm.nih.gov/). Protein motifs were analyzed using the online tool meme (https://meme-suite.org/meme/tools/meme), with 10 motifs and default parameters (Bailey et al., 2015). The gene structure, conserved domain, and motif visualization of *PRXs* genes were performed using TBtools v1.106 (Chen et al., 2020). The MEGA X alignment output results were trimmed using TrimAL v1.4.rev22 (http://trimal.cgenomics.org) with default parameters, and a visual representation was created using WebLogo 3 (https://weblogo.threeplusone.com/) (Crooks et al., 2004; Capella-Gutiérrez et al., 2009). A collinearity analysis of pepper (Zunla-1 and Chiltepin) was performed using MCScanX and visualized via TBtools (Wang et al., 2013). The pepper plant materials, which included 40-day-old seedlings, were treated with NaCl (200 mM), and the treatment was subsequently applied to the roots and leaves. The gene expression network data and stress treatment were referenced from the study by Liu et al., and the visualization of gene expression data was performed using TBtools (Liu et al., 2017).

### Pepper cDNA synthesis and quantitative real-time polymerase chain reaction analysis

The transcriptome samples designated for RNA sequencing underwent cDNA preparation. The quality of the RNA samples was confirmed through agarose gel electrophoresis and OD_260_/OD_280_ evaluation. The qualified samples were subsequently used for fluorescence quantitative experimentation to corroborate the transcriptome results. Initially, cDNA synthesis was performed using pepper RNA with K1622 from Thermo Fisher. A mixture of 2 μL pepper RNA, 1 μL Oligo(dT)^18^, and 9 μL ddH_2_O was prepared via low-speed centrifugation at 65°C for 5 min, followed by cooling on ice, another round of low-speed centrifugation, and additional cooling. In the second step, 10 μL of the product from the first step was uniformly mixed with 4 μL 5 × Reaction Buffer, 1 μL RiboLock™ RNA inhibitor, 2 μL 10 mM dNTP Mix, and 1 μL RevertAid™ M-MuLV reverse transcriptase via low-speed centrifugation. The mixture was subsequently incubated at 42°C for 60 min and then at 70°C for 5 min. Sample extraction and detection were conducted using the pepper housekeeping genes Ubiquitin (GenBank: AY486137.1) and Actin (GenBank: AY572427.1).

Primer3plus (http://www.primer3plus.com/cgi-bin/dev/primer3plus.cgi) online software was used to design primers for validating genes in the pepper transcriptome (**Supplementary Table S1**). quantitative real-time polymerase chain reaction (qRT-PCR)s were performed using the SYBR Premix ExTaq protocol (TaKaRa, Japan) via an Applied Biosystems 7500 real-time PCR system (Applied Biosystems, Foster City, CA). RNA samples from various types of pepper materials were selected with three biological replicates, and triplicate experiments were conducted using independent plant materials. PCR was conducted in the following cycles: 95°C for 30 s, 95°C for 15 s, 60°C for 30 s, and finally at 72°C for 1 min for 40 cycles. The qRT-PCR results were analyzed using the 2^–△△Ct^ method (Livak and Schmittgen, 2001). The correlation between the transcriptome and qRT-PCR results was calculated using Pearson’s correlation coefficient. The fluorescence quantitative results were visualized using GraphPad Prism v6.

## Results

### Differentially expressed genes between samples

Six cDNA libraries were constructed for transcriptome sequencing and analysis. In total, 615,793,034 reads were sequenced, yielding 83.3 Gb clean data after quality control filtering, with the Q20 and Q30 values exceeding 97% and 92%, respectively (**Table 1**). The clean data were aligned with the pepper genome to identify the differentially expressed genes. This process detected 3,304 differentially expressed genes, including 947 new transcripts. Among these genes, 2,527 (including 757 new transcripts) were upregulated in the 1933A line and 777 genes (including 190 new transcripts) were upregulated in the 1933B line.

**Table 1:** Transcriptome sequencing information of pepper.

Sample	Raw reads	Clean reads	Clean bases	Q20 (%)	Q30 (%)	GC (%)
1933A-1	95,453,648	94,839,544	12.80G	97.85	93.59	42.89
1933A-2	122,369,156	121,470,776	16.46G	97.34	92.59	42.87
1933A-3	112,715,088	111,910,540	15.24G	97.56	93.02	42.98
1933B-1	75,368,098	74,848,964	10.08G	97.55	93.00	42.67
1933B-2	95,855,742	95,150,004	12.92G	97.54	92.98	42.49
1933B-3	118,395,074	117,573,206	15.80G	97.69	93.31	42.19

### Enrichment analysis of differentially expressed genes

**Figure 1:**
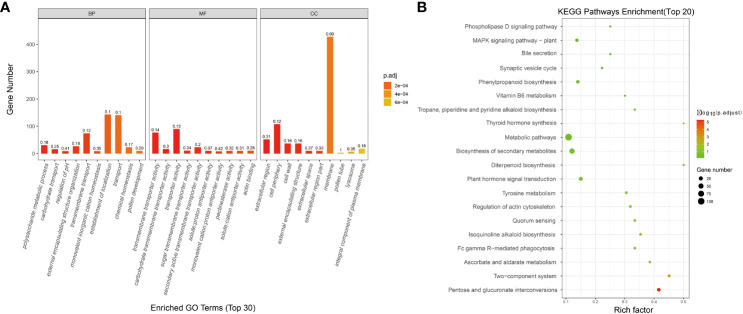
Enrichment analysis of differentially expressed genes in transcriptome, (**A** GO analysis; **B** KEGG analysis).

The transcriptome data of FL 1933A and NMSL 1933B of *Capsicum* were analyzed, and 3304 differential genes were identified. To study the biological and metabolic processes of these genes, GO and KEGG enrichment analyses were performed. In total, 1145 and 229 enriched genes were found in the GO and KEGG databases, respectively. Concerning biological processes (**Figure 1A**), 29.61% of the genes were related to the establishment of localization, 29.19% were related to transport, and 6.42% were related to metabolic processes. Meanwhile, 2.07% of the genes were related to the process of pollen development. Regarding molecular functions, 33.71% of the genes were related to transporter activity, and 28.84% were related to transmembrane transporter activity. Concerning cell composition, 60.28% of the genes were associated with the membrane, 15.21% were related to the cell periphery, 7.32% were associated with extracellular regions, 0.99% were associated with lysosomes, and 0.42% were related to pollen tubes.

The developmental process of functional pollen involves many genes and pathways. In the KEGG enrichment analysis, 157 KEGG pathways were enriched. The top five pathways were metabolic pathways, biosynthesis of secondary metabolites, plant hormone signal transduction, pentose and glucuronate interconversions, and phenylpropanoid biosynthesis. Differential gene enrichment in several metabolic processes of the NMSL and FL of pepper provided a theoretical basis for the study of pepper NMSLs (**Figure 1B**).

### Transcriptome, proteome association analysis, and candidate protein determination

**Figure 2:**
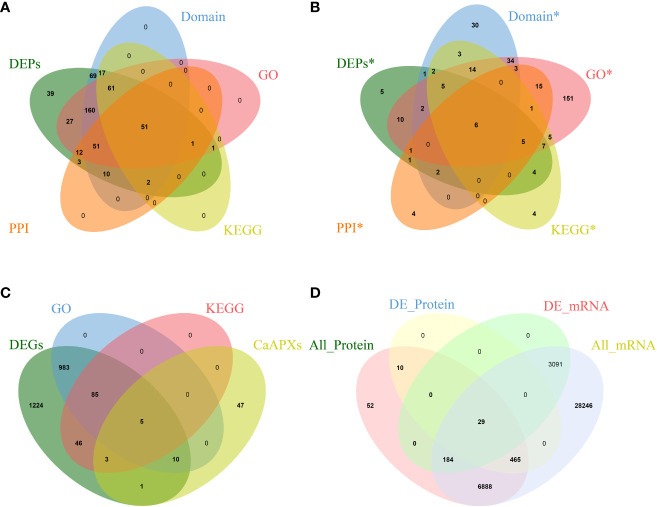
Venn diagrams of transcriptomics, proteomics, and correlation analyses, (**A** Venn diagram of proteomics; **B** Protein Venn diagram with significant difference; **C** Venn diagram of transcriptome and *CaPRX* genes family; **D** Combined analysis of Venn diagram).

Proteomic analysis of 1933A and 1933B uncovered 504 differentially expressed genes. Regarding the protein products of these genes, 351 proteins were upregulated in 1933A and 153 were upregulated in 1933B. Domain annotation, GO annotation, and KEGG annotation of these genes were performed, and the genes were annotated to 421, 364, and 133 pieces, respectively. Meanwhile, 130 proteins had protein–protein interactions, and 51 proteins were found in their intersections (**Figure 2A**). The analysis of significant differentially expressed proteins, GO and KEGG enrichment, and protein interactions in the FL and NMSL identified six candidate genes (**Figures 2A, B**) as follows: *Capana02g002746*, *Capana04g000638* (*CaPRXs*, PF00141), *Capana02g001798*, *Capana07g001786* (*CP*, PF08246; PF00112), *Capana08g001522* (*HSP70*, PF00012), and *Capana01g003855* (*LOX*, PF00305). Of these genes, *Capana02g002764* and *Capana02g001786* were upregulated in the NMSL, whereas the other four were downregulated. It is speculated that *PRXs*, *HSP70*, and *CaLOX* are important proteins involved in the NMSL process of pepper.

In total, 2357 differentially expressed genes were identified via differential gene expression analysis, of which 1083 and 139 were significantly enriched in GO and KEGG pathways, respectively. Intersection analysis with *PRX* genes revealed that 19 genes overlapped with *CaPRX* genes (**Figure 2C**). Using integrated transcriptomics and proteomics, we conducted a comprehensive analysis of proteins and genes correlations in terms of quantification and differentiation at significant levels. This analysis identified 7,628 quantifiable proteins in the FL and NMSL, with 504 proteins exhibiting statistical significance. Additionally, 38,903 genes were identified, 3,304 of which exhibited significant differences. The most substantial correlation was observed among 29 proteins and genes, and within this correlation, three specific genes, *Capana02g002746*, *Capana02g002990*, and Capana04g000638, intersected with *CaPRXs* genes (**Figure 2D**).

**Figure 3:**
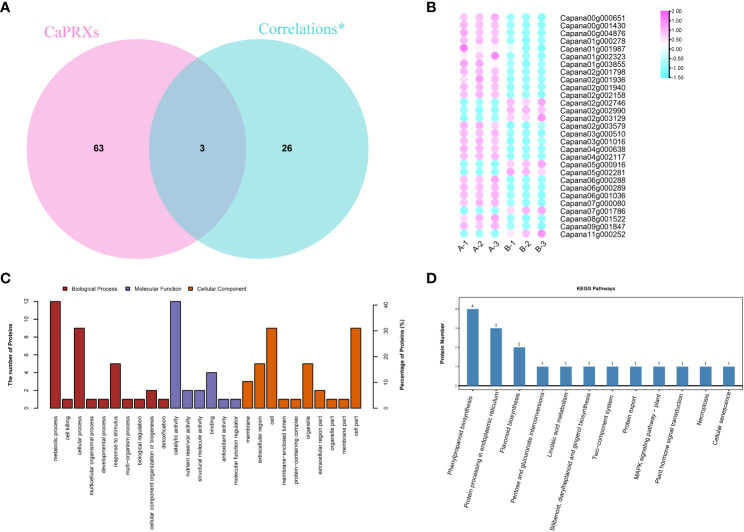
Identification and functional characterization of associated genes (**A** *CaPRX* genes family and combined analysis gene Venn diagram; **B** Combined analysis of gene expression; **C** GO comment; **D** KEGG comment).

Of the 29 significantly associated genes, three were identified as *CaPRX* genes (**Figure 3A**). Of these genes, seven proteins and genes were upregulated in the pepper NMSL and the other 22 genes were upregulated in the FL (**Figure 3B**). GO and KEGG enrichment analyses of these 29 genes were performed (**Figures 3C, D**). The results illustrated that 14 proteins were enriched in 149 biological processes, 17 proteins were enriched in 83 molecular functions, and 12 proteins were enriched in cell composition. The top three enriched biological processes were biological process, metabolic process, and organic substance metabolic process. The top three molecular functions were molecular function, catalytic activity, and hydrolase activity. The top three enriched cell components were cellular component, cell, and cell part. Meanwhile, proteomic differences in protein concentration, pollen development, pollen wall assembly, hydrogen peroxide metabolic process, and gametophyte development were also enriched. These results indicate that in GO enrichment, more genes were involved in the response to pollen development in pepper.

Two of the six key proteins identified using proteomics were PRX protein (capan02g002746 and capan04g000638). Five of the key genes identified using transcriptomics were *PRX* genes (*capan01g004380*, *Capana03g000271*, *Capana03g003406*, *Capana04g002827*, and *Capana11g001222*). Transcriptomic and proteomic association analysis identified three significant associated genes, which are presumed to be the key candidate genes involved in the regulation of male sterility in pepper (*Capan02g002746*, *Capan02g002990*, and *Capan04g000638*).

### *CaPRX*s identification and phylogenetic analysis

**Figure 4:**
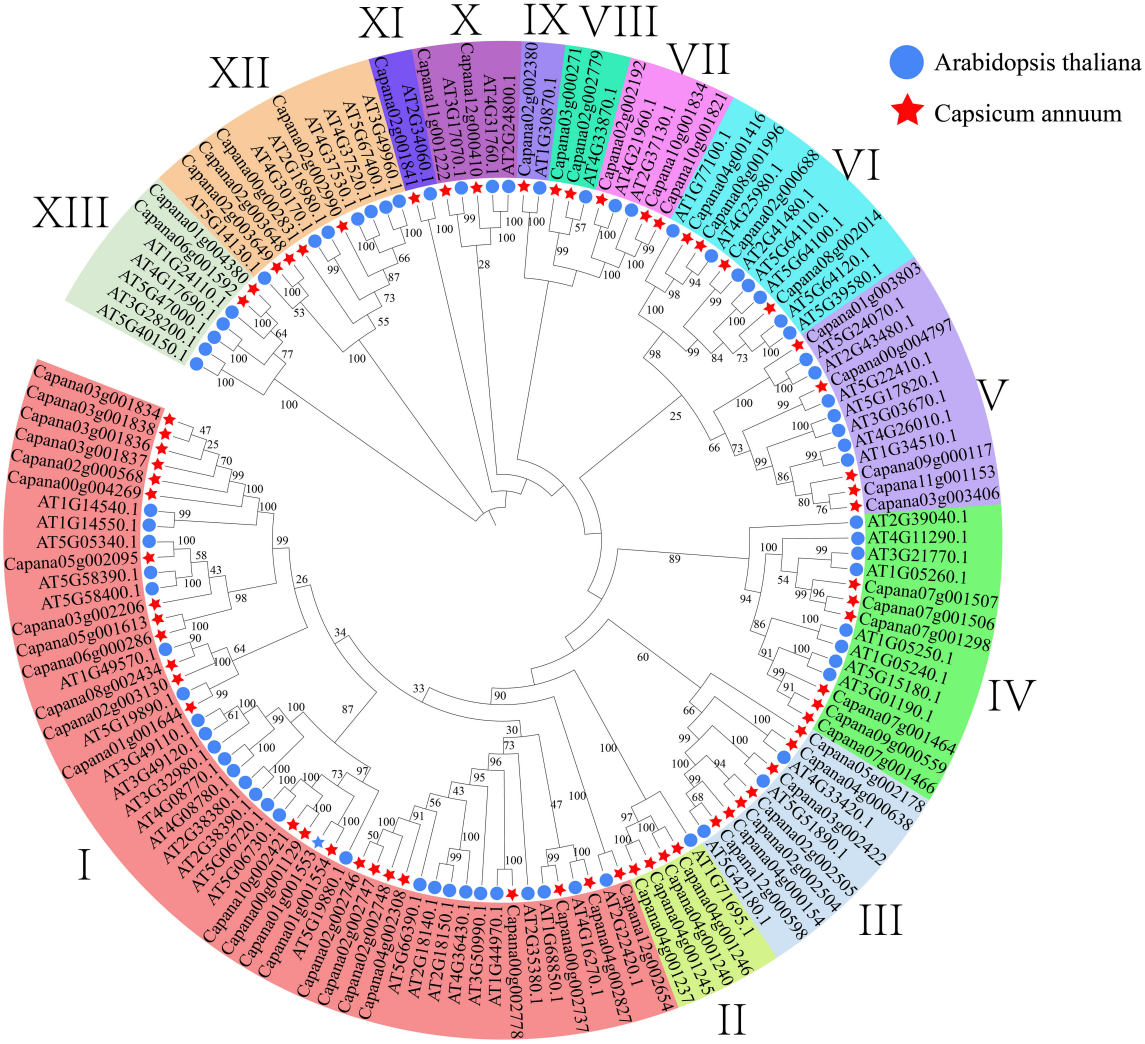
Phylogenetic tree of *CaPRX* genes in pepper (pentagons: pepper; circles: *Arabidopsis*).

Through searching the pepper whole genome using Pfam model files, we successfully retrieved 107 sequences. Subsequently, we conducted a blast comparison by aligning these sequences with *Arabidopsis AtPRX* sequences, thereby discovering 66 sequences at their intersection. These 66 sequences were identified as candidate genes in subsequent analyses and were named *CaPRX*s based on their chromosome location and gene abbreviation. To investigate the evolutionary aspects of the pepper CaPRX gene family, we constructed a comprehensive phylogenetic tree by merging *Arabidopsis AtPRX* sequences with pepper *CaPRX* sequences, which revealed that pepper can be categorized into 13 subfamilies. Notably, the largest subfamily I contained 25 CaPRX genes, whereas the smallest subfamilies IX and XI each comprised only one *CaPRX* sequence (**Figure 4**). In contrast to the results of Yang et al.’s study on *ClPRX* identification in watermelon, which reported a total of 79 gene sequences categorized into 7 subfamilies and 11 subclasses, our study, focusing on pepper, identified a total of 66 sequences distributed across 13 subclasses (Yang et al., 2022).

### Analysis of *CaPRX* gene family characteristics

An analysis of the physicochemical properties of the 66 identified CaPRX proteins yielded several interesting findings (**Supplementary Table S2**). The amino acid number within these genes varied from 222 to 493 and their molecular weights ranged from 24.84 to 53.63 kDa. The isoelectric point covered a spectrum from 4.45 to 9.85, indicating that CaPRX proteins exhibited amphipathic characteristics. The instability coefficient spanned from 21.73 to 53.08, suggesting that CaPRX proteins fell within a range of stability, with 21 proteins exhibitng an instability index of >40, thereby signifying their predicted instability. Conversely, the remaining 45 proteins demonstrated an instability index of <40, indicating their predicted stability. The hydrophilic–hydrophobic balance extended from −0.453 to 0.084, with only CaPRX19, CaPRX55, CaPRX45, CaPRX28, and CaPRX48 being classified as hydrophobic proteins, while the remaining CaPRXs exhibited hydrophilic properties.

**Figure 5:**
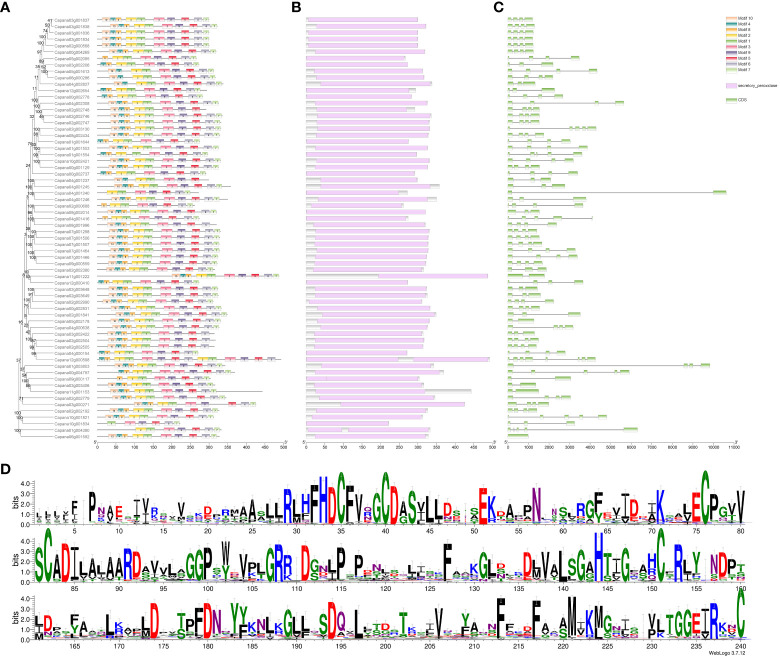
Characteristic analysis of the pepper *CaPRX* genes family (**A** Motif features; **B** Conserved domains; **C** Gene structure; **D** Sequence alignment results).

Additionally, our investigation unveiled several motifs in the CaPRX proteins in peppers, with the majority containing 10 motifs. Notably, Motifs 3 and 9 were present in all the sequences. To further analyze the conserved domains, we observed that all the CaPRX proteins featured the highly conserved domain PRXs (PF00141). Gene structures were also visualized, revealing a range of exon numbers from 1 to 8 (**Figure 5**).

### Analysis of *CaPRX*s chromosome location, collinearity, and expression characteristics

**Figure 6:**
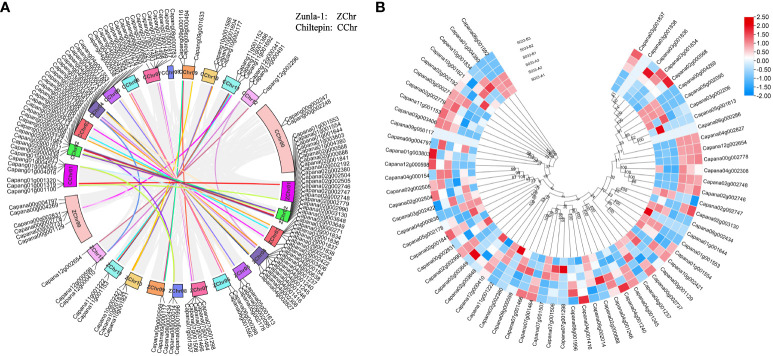
Analysis of chromosomal localization, collinearity, and expression characteristics of *CaPRX genes* in pepper (**A** Chromosomal localization and collinearity blocks of the *CaPRX genes*; **B** Expression patterns of *CaPRXs* in FL and NMSL).

The chromosomal distribution of *CaPRX* genes in peppers was notably concentrated on chromosome Chr01, containing a total of 15 *CaPRX* genes, while the least representation was observed on chromosomes Chr06, Chr09, and Chr11, each containing only 2 *CaPRX* genes (**Figure 6**). This distribution pattern suggests that *CaPRX* genes are not evenly distributed across different chromosomes, with each chromosome containing at least one *CaPRX* target gene. Remarkably, six genes were identified on the Chr00 (Genes that are not attached to chromosomes) sequence chromosome. In the genomes of Zunla-1 and wild Chiltepin, collinear blocks were observed on each chromosome, with the exception of the virtual chromosomes Chr00 and Chr08, where no collinear blocks were detected. The analysis of collinear blocks revealed that 58 collinear genes in Zunla-1 and Chiltepin share a common ancestor. Furthermore, a more in-depth examination of the *CaPRX* genes in different pepper types revealed that they were predominantly upregulated in the 1933A line. Conversely, in the 1933B line, *Capana02g002746* (*CaPRX13*) was upregulated, and *Capana04g000638* (*CaPRX30*) was upregulated in the 1933A line. Notably, these two genes displayed a reverse expression pattern, adding an intriguing dimension to the analysis.

### Salt stress treatment and qRT-PCR analysis

**Figure 7:**
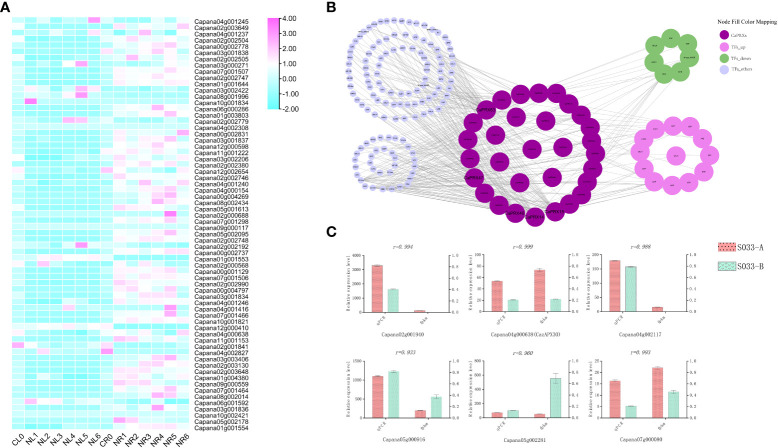
Expression characteristics, network module analysis, and qRT-PCR analysis of *CaPRXs* under salt stress (**A** Expression characteristics analysis of leaves and roots under salt stress; **B** Network module analysis under salt stress; **C** qRT-PCR analysis under salt stress).

Studies have observed that overexpression of *CaPRX* genes can promote salt tolerance in transgenic plants. To study the regulatory pathway of *CaPRX* genes in pepper, 66 *CaPRX* genes were analyzed under salt stress treatment. An analysis of tissue-specific expression patterns of *CaPRX* genes under salt stress treatment (**Figure 7A**) revealed that all 66 *CaPRX* genes exhibited varying degrees of upregulation in the roots. Conversely, their expression levels in the leaves were predominantly downregulated compared with those in the roots. These findings underscore the significant role of *CaPRXs* in the stress response of pepper roots under salt stress conditions. To investigate the expression characteristics of *CaPRXs* under salt stress, 178 network nodes and 378 network pairs were extracted from the coexpression network module (**Figure 7B**). Notably, among the genes with a degree of >10, five *CaPRX* genes were identified, namely, *CaPRX14* (degree = 41), *CaPRX15* (degree = 41), *CaPRX46* (degree = 45), *CaPRX47* (degree = 26), and *CaPRX63* (degree = 64). *CaPRX15* and *CaPRX63* were upregulated in the NMSL, while *CaPRX47* was upregulated in the FL. Within the coexpression network, the top five transcription factors were bHLH (18), ERF (14), LBD (13), MYB (12), and WRKY (12). The transcription factor with a degree of >20 was G2-like (degree = 22, *Capana00g004643*). In the transcriptome analysis, 13 transcription factors were upregulated, while 7 were downregulated in the sterile line. Six genes were randomly selected from the transcriptome, including one gene (*Capana04g000638*, *CaPRX30*) from the *CaPRX* gene family. The qRT-PCR results of the randomly selected six genes revealed a strong correlation with the transcriptome fragments per kilobase of transcript per million mapped reads results (PCCs > 0.9; **Figure 7C**). Among these genes, *CaPRX30* and *Capana07g000080* exhibited high expression levels in the NMSL, while the other four genes were upregulated in the FL.

## Discussion

Owing to the continuous innovation and advancements in breeding methods, numerous novel germplasms and resources have been developed for various economic crops, effectively ensuring agricultural and food production. The MS mutant 1933B of the pepper plant, which lacks pollen, plays a crucial role in the hybrid breeding of pepper. Herein, we conducted a combined analysis of the pepper 1933A FL and 1933B NMSL at the molecular biology and protein levels. The transcriptome analysis identified 3,304 differentially expressed genes, and GO was primarily enriched in processes related to polysaccharide metabolism, carbohydrates, pH regulation, pollen development, transmembrane transport protein activity, extracellular zone, and pollen tubes. In the KO analysis, pathways such as the phospholipase D signaling pathway, MAPK signaling pathway in plants, bile secretion, synaptic vesicle cycle, and phenylpropanoid biosynthesis pathways were enriched. These findings in GO enrichment, particularly those related to pollen development and pollen tubes, underscore the significant influence of pollen development on male sterility in pepper. MS eggplants reportedly exhibit differentially expressed genes involved in redox, carbohydrate, and amino acid metabolism (Yang et al., 2018), whereas MS watermelons and broccoli have exhibited the involvement of phenylpropanoid biosynthesis and plant hormone signaling pathways (Shu et al., 2018; Wang et al., 2020). These differentially expressed genes may play direct or indirect roles in plant pollen development processes, including meiosis and cell wall formation, thereby affecting male sterility in pepper.

In the previous proteomic studies of pepper, 1645 differentially abundant proteins were found via proteomic analysis, 45 of which were related to the anther and pollen development of MS lines of pepper, indicating that many proteins were involved in the flowering process of pepper (Pei et al., 2022). Mutations in different strains can result from various factors, including natural environmental influences or external environmental conditions. For instance, MS lines have been observed in several crops, such as rice, and its presence has been associated with temperature sensitivity. Additionally, there have been instances of light-sensitive male sterility in rice plants (Fan et al., 2016). Wu et al. (2022) found a natural allele OsMS1 that can respond to temperature changes and imparts heat-sensitive male sterility. In the model plant *Arabidopsis*, *Lox3* and *Lox4* are pivotal for male fertility and play a significant role in pollen development (Caldelari et al., 2011). During our study, peppers were cultivated in Zunyi, Guizhou Province, an area with limited light and unstable temperatures from March to April. It is hypothesized that the low temperature and insufficient light during the planting process affected the growth and development of the peppers.

Herein, we discovered natural mutations leading to male sterility and identified candidate key genes, *CaPRXs*, involved in regulating male sterility in pepper through multiomics analysis. Our analysis delved into the structural, tissue-specific, and expression characteristics of *CaPRXs* genes, revealing a total of 66 Class III peroxidases in pepper with typical PRX conserved domains. Notably, we observed tissue-specific expression in roots and leaves under salt stress, with *CaPRXs* primarily upregulated in the roots. Coexpression network analysis revealed that *CaPRXs* are coexpressed with multiple transcription factors. This finding indicates that *CaPRX*, identified via proteomics and transcriptomics in multiomics analysis, indirectly influence the process of male sterility in pepper. Furthermore, Dong et al. identified *CaMYB80* as a key regulator gene specifically expressed in anthers, with its downregulation causing male sterility in pepper (Dong et al., 2022). In wheat genetic breeding studies, *CaPRXs* were found to be upregulated during early pollen development (Hiraga et al., 2001; Liu et al., 2018). It is speculated that *CaPRX* genes are involved in the development of pepper pollen, which leads to its hypoplasia and the formation of MS line of pepper. Meanwhile, in this study, it was found that *CaPRX* genes co-express with several transcription factors via a co-expression network. Transcription factors such as bHLH, ERF, LBD, MYB, WRKY, and G2-like may play a role in male sterility of pepper via the indirect regulation of *CaPRXs*.

Numerous studies have reported that Class III peroxidases are integral to plant growth, development, and physiological processes, especially for pollen production and stress responses. For example, in a study on male sterility in tomatoes, higher overall peroxidase activity was observed in MS stamens compared with that in male fertile stamens (Markova et al., 1992). Chen et al. cloned the Ghpod gene, which contained a conserved domain of Class III peroxidase, and found its expression in the reproductive organs of cotton, particularly pollen, suggesting the involvement of peroxidases in the male reproductive process of angiosperms (Chen et al., 2009). Similarly, hazelnut (*Corylus avellana*) specifically expresses *CavPrx* in pistils, indicating the crucial role of *CaPRXs* in causing male sterility in pepper. Moreover, *PRXs* serve vital functions in various plant physiological and developmental processes, acting as essential defenses against biotic and abiotic stresses (Kidwai et al., 2020). For example, *PRXs* are not only upregulated in pollen and pistils but also participate in nonbiological stress responses. Research has found that an example includes PRXs overexpression in wheat, which increases the salt tolerance of transgenic wheat (Su et al., 2020). Our study on pepper under salt stress revealed that *CaPRXs* are involved in abiotic stress responses and were upregulated in the roots, reaffirming the diverse roles of *PRXs* in plants.

The continuous advancement of biotechnology enables its increasing practical application. For instance, Jung et al. employed gene editing to create a MS phenotype by knocking out the SlMS10 gene encoding a bHLH transcription factor (Jung et al., 2020). Similarly, Du et al. utilized CRISPR/Cas9 gene editing technology to establish a specific MS tomato line and developed a fertility restoration system using this MS line (Du et al., 2020). It is anticipated that as research on male sterility in peppers progresses, biotechnology may offer innovative germplasm solutions.

## Conclusions

This study conducted proteomics, transcriptomics, and association analyses to identify 29 key genes, highlighting a significant number of *CaPRX* genes in differential proteins and genes. The association analysis unveiled three intersections between the 29 genes and 66 *CaPRX* genes, underscoring the significant role of *CaPRXs* in male sterility in pepper and their involvement in the process. To explore the regulatory roles of *CaPRXs*, the study comprehensively identified pepper *CaPRXs* at the whole-genome level, revealing a total of 66 genes containing typical Class III peroxidase domains. The 66 *CaPRX* genes were categorized into 13 subfamilies, which were stable proteins. Salt stress showed that *CaPRXs* were mainly upregulated in roots and coexpressed with multiple transcription factors. This study speculated that the *CaPRXs* gene is involved in the development process of pepper NMSL, which is regulated by the *CaPRXs* gene, resulting in pollen agenesis of pepper NMSL. By exploring NMSL in pepper at the transcriptomic and proteomic levels, this research furnishes a theoretical foundation for pepper genetic breeding and offers practical insights for innovative breeding solutions.

## Data availability statement

The original contributions presented in the study are included in the article/**Supplementary Material**. Further inquiries can be directed to the corresponding authors.

## Author contributions

CQ: Funding acquisition, Writing – original draft, Writing – review & editing. SY: Visualization, Writing – original draft, Writing – review & editing, Software. XL: Writing – original draft, Software, Visualization. JJ: Data curation, Writing – review & editing. YG: Data curation, Writing – review & editing. LZ: Data curation, Writing – review & editing. JL: Data curation, Software, Writing – review & editing. ST: Data curation, Writing – review & editing. YL: Data curation, Writing – review & editing. TL: Data curation, Writing – review & editing. XC: Data curation, Writing – review & editing. YW: Writing – review & editing.
